# Hypoglossal Nerve Transection Induces Anxiety- and Depression-like Behaviors with HPA Axis Dysregulation in Rats

**DOI:** 10.3390/bioengineering13040425

**Published:** 2026-04-06

**Authors:** Sena Chung, Jong-Ho Lee, Doyun Kim, NaRi Seo, Bongju Kim, Jeong Won Jahng

**Affiliations:** 1 Department of Oral and Maxillofacial Surgery, School of Dentistry and Dental Research Institute, Seoul National University, Seoul 03080, Republic of Korea; s_e_na@snu.ac.kr (S.C.); leejongh@snu.ac.kr (J.-H.L.); aka.darkwood@gmail.com (D.K.); tj0943@snu.ac.kr (N.S.); 2 Department of Neurobiology and Physiology, School of Dentistry and Dental Research Institute, Seoul National University, Seoul 03080, Republic of Korea; 3 Program in Neuroscience, Department of Dental Science, Graduate School, Seoul National University, Seoul 03080, Republic of Korea; 4 Dental Life Science Research Institute, Seoul National University Dental Hospital, Seoul 03080, Republic of Korea

**Keywords:** hippocampus, hypoglossal nerve, psycho-emotional disorder

## Abstract

This study investigated whether tongue motor loss induced by bilateral transection of the hypoglossal nerves (Hx) alters anxiety- and/or depression-like behaviors in rats and examined the associated neuroendocrine changes. Male Sprague–Dawley rats underwent Hx or sham surgery and were evaluated in the ambulatory activity, elevated plus maze, forced swim, and sucrose preference tests at different postoperative time points. Neuroendocrine parameters were assessed by plasma corticosterone assay, quantitative real-time PCR, Western blot analysis, and adrenal histology. At two weeks after surgery, Hx rats exhibited anxiety-like behavioral changes in the elevated plus maze and increased immobility with reduced struggling in the forced swim test, consistent with a depression-like behavior. Reduced sucrose intake was observed at earlier postoperative stages, suggesting early anhedonia-like behavior. Hx rats also showed chronically increased plasma corticosterone levels, adrenocortical hypertrophy, and decreased hippocampal glucocorticoid receptor expression. These findings highlight a potential oral–systemic interaction in which loss of oral motor function alters neuroendocrine homeostasis and emotional regulation.

## 1. Introduction

Oral sensory and motor inputs may be disrupted by various clinical conditions, including dental surgical procedures, trauma, radiation exposure, chemotherapy, or infection. These inputs play an important role in maintaining normal brain function, particularly within the hippocampus [1]. Continuous sensory feedback from mastication has been suggested to support hippocampus-dependent processes, whereas impairment of oral motor activity is associated with structural and functional alterations in this region, ultimately leading to deficits in learning and memory [2,3,4]. For effective mastication and deglutition, tongue movements are essential. The hypoglossal nerve (cranial nerve XII) is responsible for motor control of the tongue [5,6,7], and its injury leads to impairments in mastication and food intake. We previously reported that tongue motor loss induced by bilateral transection of the hypoglossal nerves (Hx) impairs hippocampus-dependent cognitive function and induces anatomical and functional alterations in the hippocampus in rats [2]. Hippocampal function is implicated not only in cognitive behaviors but also in psycho-emotional behaviors, such as depression and anxiety [3,4]. Rats with oral sensory deficits from lingual nerve damage showed anxiety- and depression-like behaviors with hippocampal dysfunction [5]. Despite accumulating evidence linking oral function to hippocampal-dependent cognition, its role in affective regulation and neuroendocrine control remains less well understood. In particular, it is unclear whether disruption of oral motor function can influence emotional behaviors through stress-related neuroendocrine pathways such as the HPA axis. Addressing this gap may provide insight into how peripheral dysfunction in the oral system contributes to central mechanisms underlying anxiety and depression. Therefore, the present study aimed to investigate whether tongue motor loss induced by Hx leads to anxiety- and depression-like behaviors and to examine the associated neuroendocrine mechanisms.

## 2. Materials and Methods

### 2.1. Animals

Male Sprague-Dawley rats (200–250 g) were obtained from Orient bio Co. (Seongnam, Republic of Korea) and housed under controlled environmental conditions (22 ± 1 °C, 55% humidity, 12 h light/dark cycle; lights on at 07:00) at the Seoul National University Animal Facility. Animals had free access to standard laboratory chow and membrane-filtered water throughout the experimental period. All experimental procedures were conducted in accordance with the guidelines established by the Korean Academy of Medical Sciences and were consistent with the National Institutes of Health Guide for the Care and Use of Laboratory Animals. The study protocol was approved by the Institutional Animal Care and Use Committee of Seoul National University (Approval number: SNU-150310-1-1, approved on 07.01.2016). Animals were allocated to experimental groups at the cage level to prevent cross-housing between different surgical conditions. No formal randomization procedure was applied. No a priori inclusion or exclusion criteria were prespecified, and all animals that completed the experiment were included in the analysis.

### 2.2. Surgery

Rats were anesthetized via intraperitoneal injection of 3 mL/kg chloral hydrate and 45 mg/kg sodium pentobarbital. After achieving adequate anesthesia, animals were positioned on a surgical platform equipped with a non-traumatic head holder. The surgical area was prepared by trimming hair, and 10% povidone-iodine was applied. A ventral midline incision (approximately 1.5–2.0 cm) in the submandibular region was made. The digastric and masseter muscles were gently separated to expose the hypoglossal nerve and its bifurcation into medial and lateral branches. Bilateral transection was performed by removing approximately 0.5 cm segments from both branches using microfine forceps. Complete transection was confirmed by visualizing both proximal and distal nerve ends. The incision was closed using 4-0 nylon sutures in a single layer. Sham-operated animals underwent identical procedures without nerve transection. Postoperative recovery was monitored by assessing body weight and food intake.

### 2.3. Ambulatory Activity Test

Locomotor behavior was evaluated using an automated activity monitoring system on postoperative days (POD) 7 and 17. Individual rats were introduced into the recording chamber, and movement was recorded based on beam interruptions at 5 min intervals over a 60 min session. In addition to total activity counts, behaviors such as rearing, grooming, and defecation were evaluated as previously described [6]. Grooming behavior was further categorized into rostral (forepaw and head) and caudal (body, limbs, and tail/genital) components [7]. To minimize potential confounding effects of residual olfactory cues, the chamber was cleaned with 70% ethanol between sessions.

### 2.4. Elevated Plus Maze Test

Anxiety-like behavior was evaluated using the elevated plus maze on POD7 and 14. The apparatus consisted of two open arms and two enclosed arms extending from a central platform, elevated 50 cm above the floor. Each rat was placed on the central platform at the beginning of the test, and behavioral activity was recorded for 5 min. Arm entry was defined as the placement of all four paws within a given arm. Time spent in each arm and the number of entries were quantified as previously described [6,8]. The apparatus was cleaned with 70% ethanol between trials.

### 2.5. Forced Swim Test

Depression-like behavior was evaluated using the forced swim test at POD10 and 17. Rats were placed in a cylindrical container filled with water, and their behavior was recorded over a 5 min period. Immobility was defined as the minimal movement required to maintain the head above water. Swimming was defined as active movement exceeding that required to maintain the head above water, and struggling as vigorous climbing movements directed against the walls of the container, as previously described [6,9]. Behavioral scoring was conducted using video recordings. All behavioral tests were performed between 09:00 and 12:00 to minimize circadian influences, and observers were blinded to experimental groups.

### 2.6. Sucrose Preference Test

Anhedonia-like behavior was assessed using a sucrose preference paradigm beginning at POD9. Rats were given simultaneous access to two bottles containing either 5% sucrose solution or water for 1 h daily over 7 consecutive days. To avoid positional bias, bottle placement was alternated each day. Prior to testing, animals underwent 20 h of water deprivation, followed by 3 h of water access after each session.

### 2.7. Plasma Corticosterone Assay

Blood samples were obtained from the tail vein at POD1, 7, 15, and 21. Following centrifugation at 2000 rpm for 20 min at 4 °C, plasma was collected and stored at −80 °C until analysis. Plasma corticosterone levels were measured using a commercial enzyme immunoassay kit. All sampling procedures were conducted between 09:00 and 11:00 to minimize circadian variation.

### 2.8. Histology of the Adrenal Gland

At POD15, animals were deeply anesthetized with 65 mg/kg sodium pentobarbital and subjected to transcardial perfusion using heparinized phosphate-buffered saline, followed by fixation with ice-cold 4% paraformaldehyde in 1X PBS. Adrenal glands were collected, weighed, and post-fixed for 2 h, then cryoprotected in 30% sucrose for 24 h. Tissue sections were prepared using a cryostat and stained with hematoxylin and eosin following standard procedures.

### 2.9. Quantitative Real-Time PCR (qRT-PCR)

Hippocampal tissues were collected at POD11 from rats not subjected to behavioral testing. Samples were rapidly dissected, snap-frozen in liquid nitrogen, and stored at −80 °C. Total RNA was isolated using TRIzol reagent (15596018, Invitrogen, Carlsbad, CA, USA), followed by chloroform-mediated phase separation and isopropanol precipitation. RNA pellets were washed with 80% ethanol and resuspended in RNase-free water. cDNA synthesis was performed using SuperScript II Reverse Transcriptase (#18064, Invitrogen, Carlsbad, CA, USA) with 1 μg of RNA. qRT-PCR was conducted using Power SYBR Green PCR master mix on a 7500 Gene Amp PCR system. Gene expression levels of glucocorticoid receptor (GR; forward-CCT GTT GGC ACC AGC TAT CA and reverse-GCC TAT GTA ATC TGC TCA GCC T) were normalized to GAPDH (forward-CCC TTC ATT GAC CTC AAC TAC ATG GT and reverse-CCA GCA TCA CCC CAT TTG ATG TTA) and analyzed using the comparative Ct method.

### 2.10. Western Blot Analysis

Hippocampal samples were collected at POD14 and processed for protein analysis. Tissues were homogenized in extraction buffer containing protease and phosphatase inhibitors. Following centrifugation at 13,000 rpm for 20 min at 4 °C, supernatants were collected. 40 μg proteins were separated by SDS-PAGE and transferred onto PVDF membranes. Membranes were blocked with 4% skim milk for 1 h at room temperature (RT) and then incubated with primary antibodies against GR in the blocking solution overnight at 4 °C. Membranes were incubated with horseradish peroxidase (HRP)-conjugated secondary antibodies (PI-2000, Vector Laboratories, CA, USA) for 2 h at RT. Protein bands were detected using chemiluminescence and quantified using ImageJ (ver. 1.53k). GR expression levels were normalized to β-actin.

### 2.11. Statistical Analysis

Statistical analyses were performed using GraphPad Prism version 8.4.3 (GraphPad Software, San Diego, CA, USA). Data are presented as mean ± SEM. For comparisons between two independent groups at a single time point, the Mann–Whitney U test was used. Behavioral data from the elevated plus maze and forced swim tests were analyzed using this approach. Repeated measurements over time, including body weight gain, sucrose intake, and plasma corticosterone levels, were analyzed using two-way repeated-measures ANOVA, followed by Bonferroni’s post hoc test to account for multiple comparisons. Exact sample sizes are provided in each figure legend. *p* < 0.05 was considered statistically significant.

## 3. Results

### 3.1. Body Weight Gain

**Figure 1:**
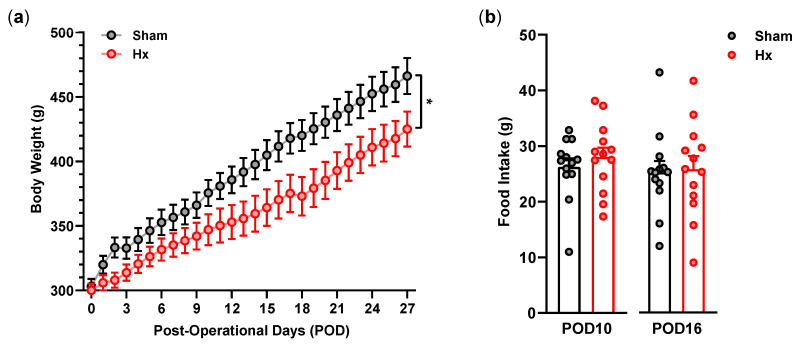
Body weight gain (**a**) and food intake (**b**) during the post-operative days (POD) (*n* = 13/group). * *p* < 0.05, two-way repeated-measures ANOVA followed by Bonferroni’s post hoc test.

Body weight gain appeared to be delayed in Hx rats compared with the sham operation group (Figure 1a). Significant differences were observed between the groups (*p* < 0.05). However, the daily chow intake measured on days 10 and 16 was not different between the groups (Figure 1b).

### 3.2. Behavioral Assessments

**Figure 2:**
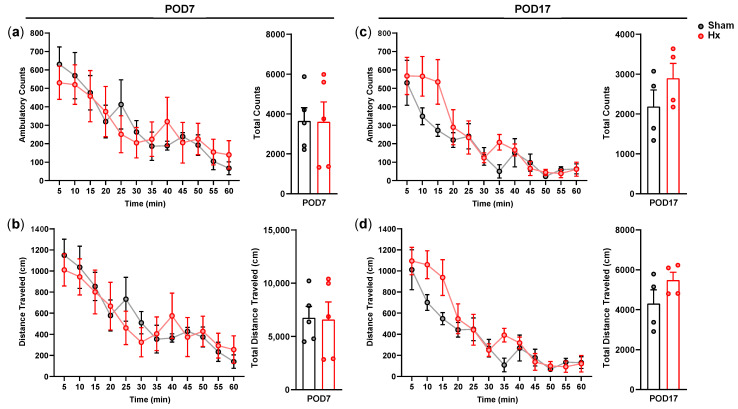
Ambulatory activity was measured for 60 min. Red lines indicate the Hx group, while gray lines indicate the Sham group. (**a**) Ambulatory counts and (**b**) distance traveled on the 7th day after the surgery (*n* = 5/group). (**c**) Ambulatory counts and (**d**) distance traveled on the 17th day after the surgery (*n* = 4/group).

Ambulatory activities of sham and Hx rats were measured for 60 min in a computerized activity chamber on the 7th or 17th day after the surgery (Figure 2). Ambulatory counts and distance traveled during the test session were not significantly different between the groups on both test days. Rearing, grooming, and defecation activities were also scored during each ambulatory activity test session, and no significant between-group differences were observed.

**Figure 3:**
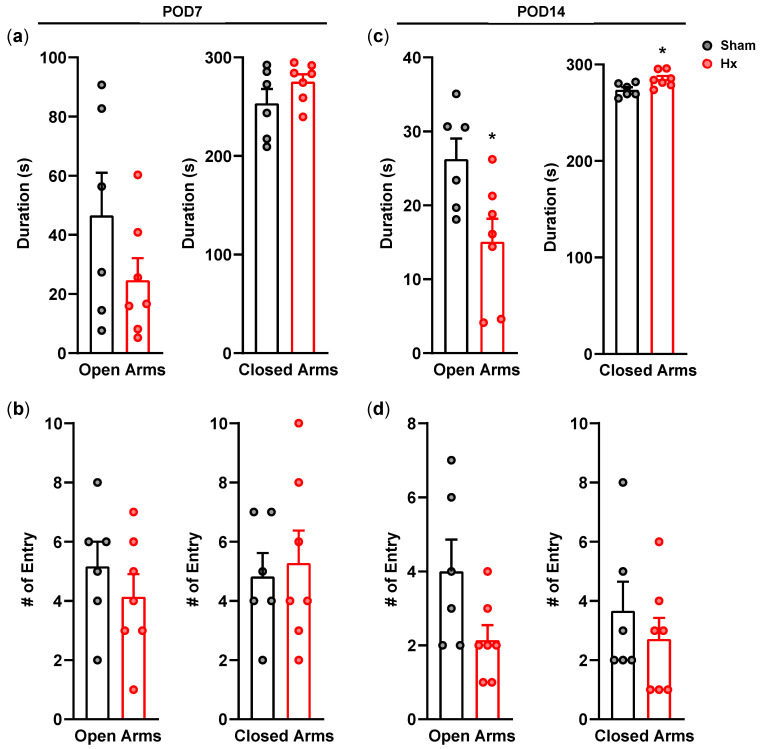
Elevated plus maze test. (**a**) Time spent in the open or the closed arms and (**b**) the number of entries to the open or to the closed arms during 5 min of the test period on the 7th day after the surgery (Sham: *n* = 6 and Hx: *n* = 7). (**c**) Time spent in the open or the closed arms and (**d**) the number of entries to the open or to the closed arms during 5 min of the test period on the 14th day after the surgery (Sham: *n* = 6 and Hx: *n* = 7). * *p* < 0.05, Mann–Whitney test.

When rats were subjected to the elevated plus maze test on postoperative day 7, time spent (Figure 3a) and the number of entries into the open arms (Figure 3b) during the 5 min test session tended to decrease in Hx rats compared with sham rats, although the difference did not reach statistical significance. However, on postoperative day 14, Hx rats spent significantly less time in the open arms and more time in the closed arms than sham rats (Figure 3c; *p* < 0.05), and the number of entries into the open arms tended to decrease in Hx rats (Figure 3d).

**Figure 4:**
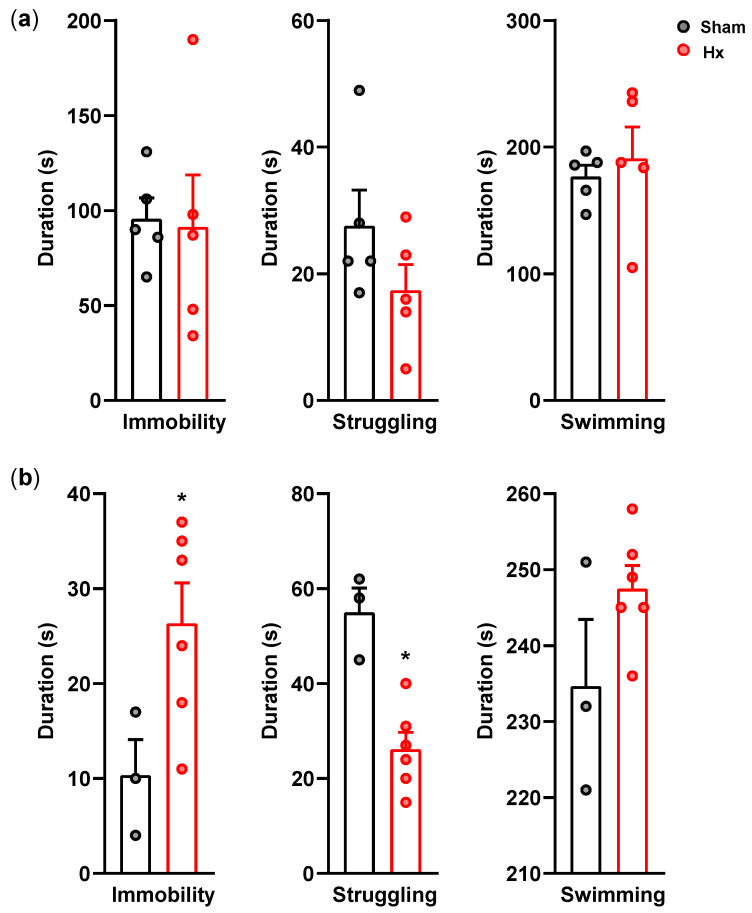
Forced swim test performed after the elevated plus maze test at 10th ((**a**); *n* = 5/group) or the 17th day ((**b**); Sham: *n* = 3 and Hx: *n* = 6) after the surgery. * *p* < 0.05, Mann–Whitney test.

During the forced swim test performed on postoperative day 10, none of the measured behavioral parameters differed significantly between Hx and sham rats (Figure 4a). However, on postoperative day 17, immobility duration was significantly increased, and struggling was decreased in Hx rats compared with sham rats (*p* < 0.05) (Figure 4b).

**Figure 5:**
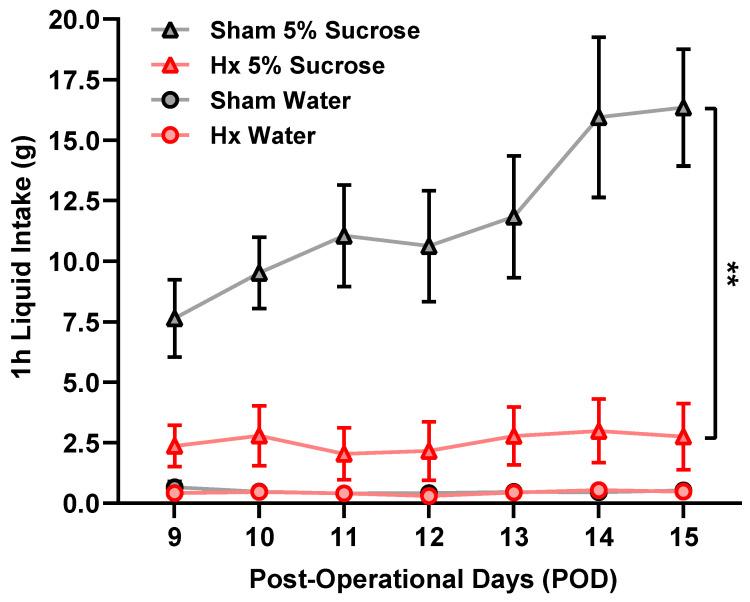
Sucrose preference test. Rats had free choices of sucrose and water during 1 h of the test period daily (*n* = 6/group). ** *p* < 0.01, two-way repeated-measures ANOVA followed by Bonferroni’s post hoc test.

Rats were subjected to the sucrose preference test from day 9 to 15 after the surgery (Figure 5). Rats had free choices of sucrose and water for 1 h daily. Sucrose, but not water, intake was significantly decreased in Hx rats compared with sham rats during each test session (*p* < 0.01), indicating the development of anhedonia-like behavior in Hx rats. Daily water intake did not differ significantly between the groups during the experimental period.

### 3.3. Plasma Corticosterone, Adrenal Gland and Glucocorticoid Receptors

**Figure 6:**
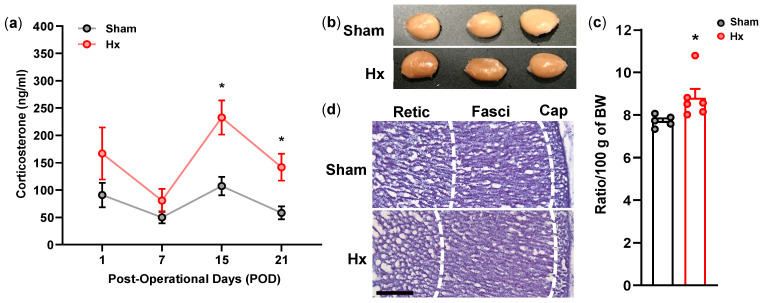
Plasma corticosterone levels at the 1st, 7th, 15th, and 21st day after the sham (*n* = 9) or Hx (*n* = 8) surgery (**a**), representative adrenal gland photos (**b**), normalized weight (**c**), Sham: *n* = 5 and Hx: *n* = 6), and H&E-stained sections of adrenal glands (**d**) at the 15th day after the surgery. * *p* < 0.05, two-way repeated-measures ANOVA followed by Bonferroni’s post hoc test or Mann–Whitney test. Scale bar: 200 μm.

Tail blood was collected on the 1st, 7th, 15th, and 21st day after the surgery. The plasma corticosterone levels of Hx rats were significantly increased on day 15 after the surgery (*p* < 0.05 vs. sham on the same day), and then decreased on day 21 compared with day 15; however, the levels remained significantly higher than those in sham rats on day 21 (*p* < 0.05) (Figure 6a).

The adrenal glands were weighed on day 15 after the surgery to examine if the chronic increase in the plasma corticosterone was accompanied by adrenal hypertrophy (Figure 6b). The absolute adrenal weight did not differ between Hx and sham rats (28.18 ± 1.30 mg in sham vs. 25.95 ± 1.59 mg in Hx). However, when normalized to body weight (adrenal weight per 100 g body weight), adrenal weight was significantly greater in Hx rats than in sham rats (*p* < 0.05; 7.72 ± 0.28 in sham vs. 8.81 ± 1.01 in Hx) (Figure 6c). In the H&E stained adrenal gland sections, zona fascicularis in the adrenal gland of Hx rats seemed to be enlarged, as compared to sham rats (Figure 6d).

**Figure 7:**
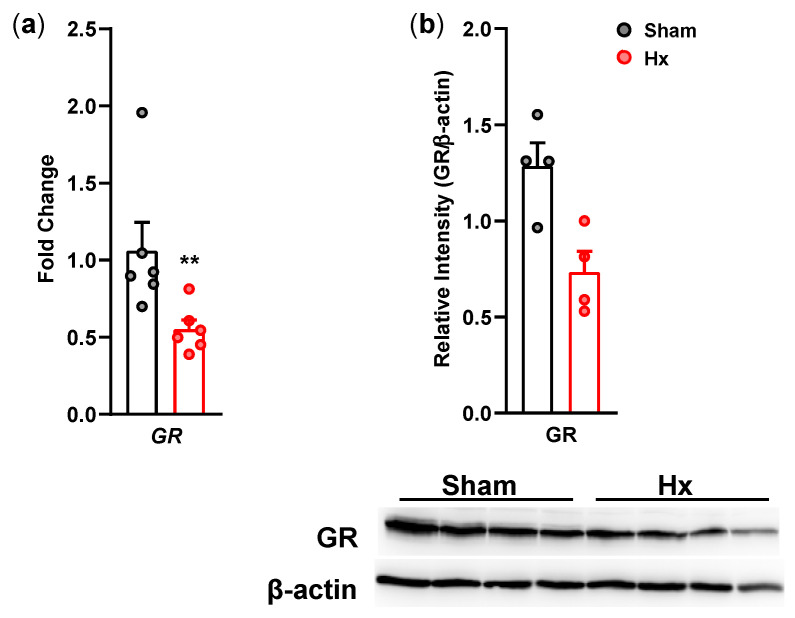
Glucocorticoid receptor mRNA ((**a**); *n* = 6/ group) and protein ((**b**); *n* = 4/ group) levels in the hippocampus on the 11th or 14th day after the surgery, respectively. ** *p* < 0.01, Mann–Whitney test.

GR mRNA (Figure 7a) and protein expression (Figure 7b) in the hippocampus were examined on day 11 or 14 after the surgery with qRT-PCR and Western Blot analyses, respectively. Hippocampal GR mRNA and protein expression levels were decreased in Hx rats compared with sham rats (*p* < 0.01).

## 4. Discussion

Tongue motor loss induced by bilateral hypoglossal nerve transection (Hx) induced anxiety- and depression-like behavioral alterations. In the elevated plus maze, Hx rats exhibited reduced exploration of the open arms together with increased occupancy of the closed arms at later postoperative stages, indicating an anxiety-like state. In the forced swim test, increased immobility accompanied by reduced struggling emerged at postoperative day (POD) 17, consistent with a depression-like or passive coping phenotype. Because overall locomotor activity remained unchanged, these behavioral alterations are unlikely to be attributable to general motor impairment. Anhedonia-like behavior, assessed by reduced sucrose intake, emerged earlier than the behavioral changes observed in the forced swim test, suggesting that anhedonia may represent an early affective alteration in this Hx model [10]. In the present study, anhedonia was observed in Hx rats from POD9 after the surgery, whereas depression-like behavior from the forced swim test was observed on POD17, but not on POD10, suggesting that anhedonia may precede more overt depression-like behavioral changes in the Hx rat model. Previous studies have suggested that decreased mastication associated with tooth loss is linked to depression and anxiety in humans [11,12,13]. The hypoglossal nerves control tongue movements and support mastication. Also, previous studies have reported that rats with oral sensory denervation showed anxiety-/depression-like behaviors [5], and an oral sensory overload increased anxiety-like behaviors in rats [14]. Collectively, the present data support a functional link between disrupted oral sensorimotor input and hippocampal dysfunction, providing a plausible basis for the observed neurobehavioral alterations.

These behavioral alterations suggest that tongue motor loss may act as a persistent physiological stressor, potentially engaging systemic stress-response pathways [15]. In particular, activation of the hypothalamic–pituitary–adrenal (HPA) axis represents a key mechanism linking peripheral dysfunction to affective regulation. Activation of this axis leads to the release of glucocorticoids, including cortisol in humans and corticosterone in rodents. Consistent with this framework, plasma corticosterone levels were elevated in Hx rats on POD15 and 21, indicating sustained activation of the HPA axis. This interpretation is further supported by adrenal cortical hypertrophy and reduced GR expression in the hippocampus. The temporal pattern of plasma corticosterone levels observed in this study may reflect a phased HPA axis response to hypoglossal nerve transection. The initial decrease from POD1 to 7 may indicate transient adaptation following acute surgical stress, whereas the subsequent increase at POD15 likely reflects sustained activation of the HPA axis driven by chronic oral motor dysfunction and associated metabolic stress. The partial normalization by POD21 may suggest compensatory negative feedback mechanisms, although the persistently elevated corticosterone levels, together with reduced hippocampal GR expression, indicate incomplete recovery of HPA axis regulation. Hyperactivation of the HPA axis is a well-recognized feature of depressive states [16,17], frequently accompanied by elevated glucocorticoid levels [18,19]. In parallel, adrenal hypertrophy is consistently observed under conditions of prolonged stress exposure [11] and in experimental models of affective disorder [12,13]. These findings suggest that HPA axis hyperactivity associated with tongue motor loss may contribute to the development of anxiety- and/or depression-like behaviors in the Hx rat model.

Beyond systemic neuroendocrine regulation, HPA axis activation is known to directly influence hippocampal function, particularly synaptic plasticity mechanisms. In addition to its role in neuroendocrine feedback regulation, GR signaling is critically involved in the modulation of hippocampal synaptic plasticity. Activity-dependent immediate early genes such as activity-regulated cytoskeleton-associated protein (Arc) play a central role in this process. Arc expression is tightly regulated by synaptic activity, particularly through NMDA receptor activation, and is rapidly induced in active neuronal circuits. Once expressed, Arc modulates dendritic spine morphology and promotes AMPA receptor endocytosis, thereby regulating synaptic strength and excitatory-inhibitory balance within neuronal networks [20]. In this context, the decreased hippocampal GR expression observed in Hx rats may reflect not only impaired HPA axis feedback but also broader dysregulation of activity-dependent synaptic plasticity mechanisms. Given that Arc functions as a key mediator of synaptic remodeling and network stability, alterations in GR signaling may indirectly influence Arc-related pathways, contributing to structural and functional changes in hippocampal circuits. Such disruptions may underlie the emergence of anxiety- and depression-like behaviors observed in this model. Although Arc expression was not directly examined in the present study, these findings suggest a plausible link between HPA axis dysregulation, impaired synaptic plasticity, and affective behavioral alterations following hypoglossal nerve transection.

Notably, while the present study primarily focused on neuroendocrine alterations associated with HPA axis dysregulation, other mechanisms, including neurotransmitter systems, such as monoaminergic and glutamatergic signaling, are also known to contribute to affective regulation and may be involved in the observed behavioral changes. It has been reported that anhedonia is accompanied by elevated corticosterone levels and adrenal hypertrophy in rat models of stress-induced depression [21,22]. In the present study, the anhedonic feature of Hx rats was observed even before the corticosterone increase. Although it would have been clear if corticosterone levels were examined with shorter time intervals between day 7 and 15 after the surgery, for now, it is likely that circulating corticosterone levels may not be closely related to anhedonic symptoms in the Hx rat model of depression. In the present study, the weight gain of Hx rats was reduced gradually compared with that of sham rats, although daily food intake did not significantly differ between the groups over the experimental period. Weight loss of Hx rats did not appear to be due to increased locomotion, because the ambulatory activity of Hx rats did not differ from that of sham rats. Weight loss was reported with elevated corticosterone levels in rat models of stress-induced depression [22,23], concurring with the present results. Adrenal glucocorticoids have been implicated in the regulation of energy homeostasis [24], and peripherally administered glucocorticoids suppress food intake and weight gain in rodents [25]. However, the weight loss of Hx rats was not accompanied by decreased food intake in the present study. It remains unclear whether the increased plasma corticosterone levels contributed to weight loss in Hx rats, because reduced body weight gain appeared to precede the increase in plasma corticosterone in the present study. It is possible that reduced body weight gain, as a metabolic stressor, may have contributed to HPA axis activation and the elevation of plasma corticosterone levels in the Hx rat model. Further studies are warranted to define the underlying mechanism by which Hx induces weight loss.

**Figure 8:**
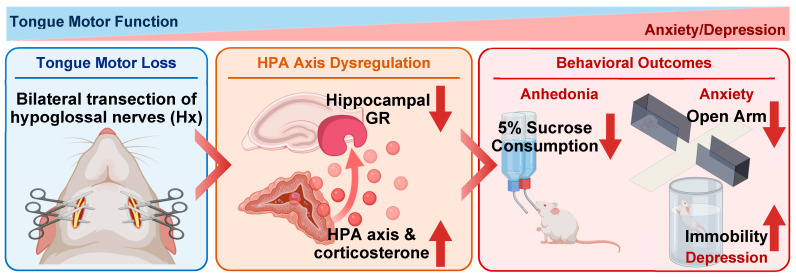
Proposed mechanism linking oral motor dysfunction to HPA axis dysregulation and affective behavioral changes. Bilateral transection of the hypoglossal nerve (Hx) induces tongue motor loss, resulting in reduced oral sensory and motor input. This alteration may act as a chronic stressor, leading to activation of the hypothalamic–pituitary–adrenal (HPA) axis and increased circulating corticosterone levels. Elevated corticosterone is associated with reduced glucocorticoid receptor (GR) expression in the hippocampus, suggesting impaired negative feedback regulation of the HPA axis. These neuroendocrine changes may contribute to hippocampal dysfunction, which in turn may underlie the development of anhedonia-, anxiety-, and depression-like behavior through decreased sucrose consumption, reduced open arm occupancy, and increased immobility time, respectively.

Taste-related sensory signals transmitted to the brainstem are not only relayed to the gustatory cortex but also distributed to multiple limbic and hypothalamic regions, including the hippocampus, amygdala, hypothalamus, and nucleus accumbens. These circuits contribute to the encoding of taste-related memory as well as affective responses such as preference and aversion. Consistent with this, oral sensory and motor inputs play an essential role in maintaining normal hippocampal function [1,26,27]. Disruption of these inputs may therefore influence hippocampal activity through multiple pathways. In addition, structural changes in the tongue epithelium following Hx could further modify sensory input; however, this possibility was not directly addressed in the present study and remains to be investigated. Taken together, the present results are consistent with our previous reports demonstrating that hypoglossal nerve transection induces hippocampal dysfunction, including decreased brain-derived neurotrophic factor (BDNF) expression, reduced neurogenesis, and impaired synaptic plasticity [2,5,14]. These findings support the notion that disrupted oral sensory and motor input may affect hippocampal structure and function, thereby contributing to the observed neurobehavioral alterations (Figure 8). Although some experiments were conducted with relatively modest sample sizes, the Hx model produces robust and reproducible phenotypic changes following a well-defined surgical manipulation, with consistent effects observed across multiple behavioral and neuroendocrine assessments within this experimental framework.

## 5. Conclusions

Bilateral hypoglossal nerve transection induced anxiety- and depression-like behavioral changes in rats, with reduced sucrose preference emerging at an earlier postoperative stage. These behavioral alterations were accompanied by increased plasma corticosterone levels, adrenal cortical changes, and reduced hippocampal glucocorticoid receptor expression, suggesting the involvement of HPA axis dysregulation. Collectively, these findings support the concept that loss of oral motor function may affect emotional regulation and neuroendocrine homeostasis. The present findings provide experimental evidence that oral motor dysfunction may influence systemic neuroendocrine regulation, highlighting the importance of considering oral–systemic interactions in the development of future diagnostic and therapeutic strategies.

## Data Availability

The datasets generated during and/or analyzed during the current study are available from the corresponding author on reasonable request.

## Abbreviations

The following abbreviations are used in this manuscript:

Hx	Bilateral transection of the hypoglossal nerves
POD	Post-operation days
Retic	Zona reticulata
Fasci	Zona fascicularis
Cap	Capsule
BW	Body weight
GR	Glucocorticoid receptor
